# Characterisation and analysis of surface integrity and residual stress in laser direct energy deposited 316 L alloy subject to plasticity ball burnishing

**DOI:** 10.1038/s41598-025-07496-3

**Published:** 2025-07-02

**Authors:** Mohammad Uddin, Joel Rech, Colin Hall, Thomas Schlaefer

**Affiliations:** 1 UniSA STEM, University of South Australia, Mawson Lakes, SA 5095 Australia; 2 ENISE, Centrale Lyon, Saint Etienne cedex 2, 42023 France; 3 Future Industries Institute, University of South Australia, Mawson Lakes, SA 5095 Australia; 4 Laserbond Ltd, Cavan, SA 5094 Australia

**Keywords:** Laser direct energy deposition, 316 alloys, Ball burnishing, Surface integrity, Residual stress, Micro-hardness, Grain modification, Metals and alloys, Mechanical engineering

## Abstract

This paper presents the effect of ball burnishing on the surface integrity and residual stress of laser-direct energy-deposited (DED) 316 L alloys, with a particular focus on surface modification characteristics across two directional planes relative to the burnishing direction. The results show that the burnishing significantly improved surface finish, reducing Ra and Sa by 76% and 51%, respectively. Additionally, the burnishing altered the grain structure from cellular/columnar to equiaxed within 50 μm deep from the top surface, with the most pronounced changes occurring in the cross-sectional plane normal to the burnishing direction. The process also converted tensile stresses into compressive stresses, with the peak compressive stress being 99% higher than that of the ground surface. Notably, the compressive stress was higher along normal to the burnishing direction compared to the burnishing direction itself. Furthermore, the burnishing increased the full width at half maximum (FWHM) by broadening X-ray diffraction (XRD) peaks, with the greatest increase observed at a depth of 68 μm, confirming the severe grain alternations. Due to grain modification and dislocation movement, the burnishing increased microhardness by 32% at the top surface, with a hardened layer extending up to 400 μm in depth. The improvement in hardness was more significant on the plane normal to the burnishing direction.

## Introduction

Laser Direct Energy Deposition (DED) is an emerging technology that builds or repairs components in aerospace, automotive, resource, and defense sectors^1^. In the DED process, metal powders are melted by a laser and rapidly cooled, forming deposited layers in an upward build direction. This intrinsic cycle of directional heating and cooling during the process leads to inhomogeneous microstructures, high surface roughness, and surface tensile stress, which can negatively impact fatigue, corrosion, and wear resistance^2^. Consequently, DEDed components are typically unsuitable for immediate use after fabrication, and therefore, often require post-processing treatments to achieve the desired surface integrity.

Surface and subsurface defects, such as surface troughs, cracks, and tensile stress, are common precursors to failure. Enhancing the surface and subsurface properties in a controlled manner can help prevent failure initiation, such as from fatigue, thereby extending the service life of the components.

Mechanical surface treatments have shown promise in addressing the surface integrity issues of additively manufactured parts^3^. Techniques such as shot peening, ultrasonic nano surface treatment (UNSM), and laser shock peening are often employed to modify microstructure, induce compressive residual stress, and increase surface hardness^4–6^. However, these methods can sometimes lead to high roughness and crack formation due to the random impact loading, which serves as a potential source for crack initiation and concentrated tensile stress, despite some benefits in subsurface grain refinement and compressive stress induction.

An alternative approach gaining traction is ball burnishing (BB), which is increasingly used to modify laser-DEDed components. BB involves plastically deforming the top surface layer by pressing a ball roller against the surface^7^. This process not only smooths the surface but also induces microstructural changes and converts tensile stress to compressive stress at deeper levels of the surface and subsurface, all of which enhance functional performance. The effectiveness of BB in improving surface integrity has been demonstrated in studies on additively manufactured AISI 431 and Stellite 21 alloys^8,9^. Furthermore, integrating ultrasonic vibration into the burnishing process significantly enhances surface integrity and functional performance—including strength, wear resistance, and fatigue resistance—compared to conventional burnishing without vibration^10,11^.

Most past research has focused on residual stress and surface profile along a specific direction, either for fixed or varying burnishing parameters. However, during burnishing, plastic deformation and material flow occur in three primary directions – longitudinal, transverse, and normal – relative to the burnishing tool movement. These directions can significantly influence the changes in surface topography, microstructure, and affected depth. The directionality of the burnishing process relative to the initially treated surface is thus an important consideration. Understanding the deformation mechanisms relative to the burnishing direction will help optimize the burnishing path strategy, yielding improved surface integrity.

In DED, laser scanning and pitch directions also influence the formation and distribution of microstructures along the build direction. However, the impact of burnishing direction on plastic deformation, grain modification, and residual stress in relation to the DED directions remains insufficiently explored in the literature.

In this context, Manjhi et al. (2024) studied parallel and cross burnishing path strategies on wire-arc additively manufactured (WAAMed) AZ31 alloy under constant force and found that the cross path led to deeper grain refinement and more surface compressive stress, but they did not explore the reasons behind this directional effect^12^. Similarly, Chomienne et al. (2016) conducted turning and burnishing on 15-5PH steel round bars, revealing variations in residual stress along the axial and circumferential directions^7^. Other studies have applied a grinding-burnishing approach to DEDed 431 alloy (martensitic steel), demonstrating its positive effects on corrosion, tribological, and fatigue properties^9,13^.

316 L alloy, austenitic steel with excellent strength and corrosion resistance, is widely used in marine and aerospace applications due to its suitability for complex geometries. Previous studies on laser DED of 316 L alloys have shown successful deposition and improved microstructure and mechanical properties^2,14^. Various laser parameters and vibration assistance during DED have been investigated to improve grain homogeneity, hardness, wear resistance^15^and corrosion resistance^16^. However, there has been little research on the ball burnishing of laser-DEDed 316 L alloys and its effects on surface integrity and residual stress, particularly with respect to burnishing and DED process directionality. Liu et al. (2024) demonstrated the use of in-situ ultrasonic roller burnishing on 316 L in L-DED, resulting in grain transformation and deeper affected depth, enhancing corrosion resistance^17^. In contrast, Luo et al. (2016) applied laser shock peening to laser-cladded 316 L, creating a deeper compressive stress layer but resulting in micro-cavities in the subsurface, which increased surface roughness^18^.

To address this gap, it is essential to explore the correlation between residual stress and the direction of plastic straining during burnishing on laser-DEDed materials. This knowledge will clarify the mechanisms behind directional performance, such as corrosion and fatigue, and enable the design of more effective surface treatment strategies.

**Fig. 1:**
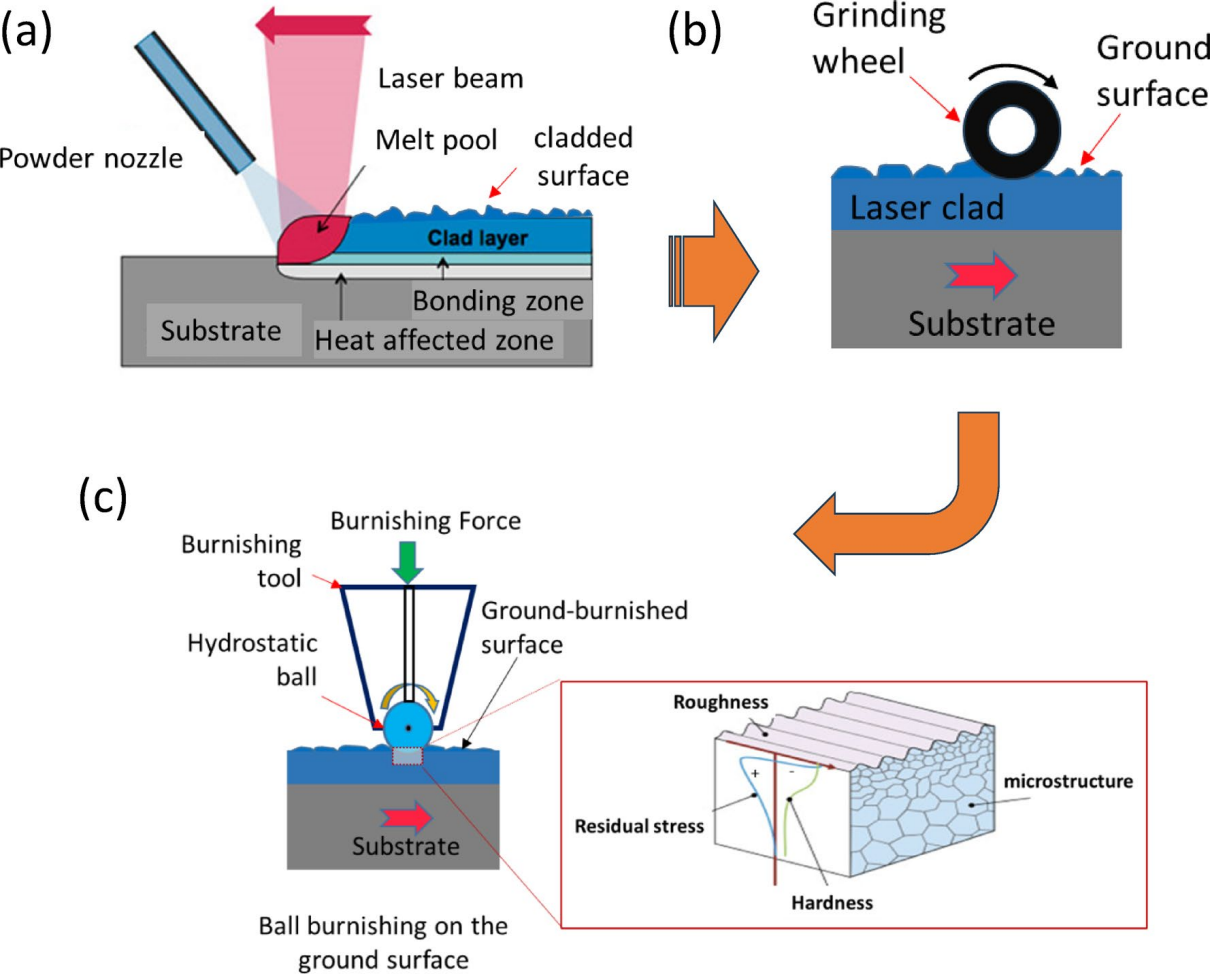
The proposed grinding-burnishing strategy applied on laser metal deposited 316 L alloy (**a**) laser deposition process (**b**) grinding on as-cladded surface and (**c**) ball burnishing on the ground surface.

This study aims to investigate the directional effects of ball burnishing (BB) on the surface integrity of laser-DEDed 316 L alloys. The treatment strategy, as illustrated in Fig. 1, involves grinding the as-DED surface followed by a ball burnishing process. The focus is on comprehensively evaluating the directional effects of this combined treatment in terms of surface roughness, in-depth microstructure, residual stress, and microhardness. To explore the directional effect, the treated specimen’s surface was metallurgically sectioned along two planes: (1) along the burnishing direction and (2) normal to the burnishing direction. Extensive characterisation using optical profilometry, SEM, XRD, and hardness testing was conducted to anayse the changes in microstructure, surface/profile residual stress, and hardness due to the burnishing directionality.

## Materials and methods

### Laser metal deposition and specimen Preparation

**Fig. 2:**
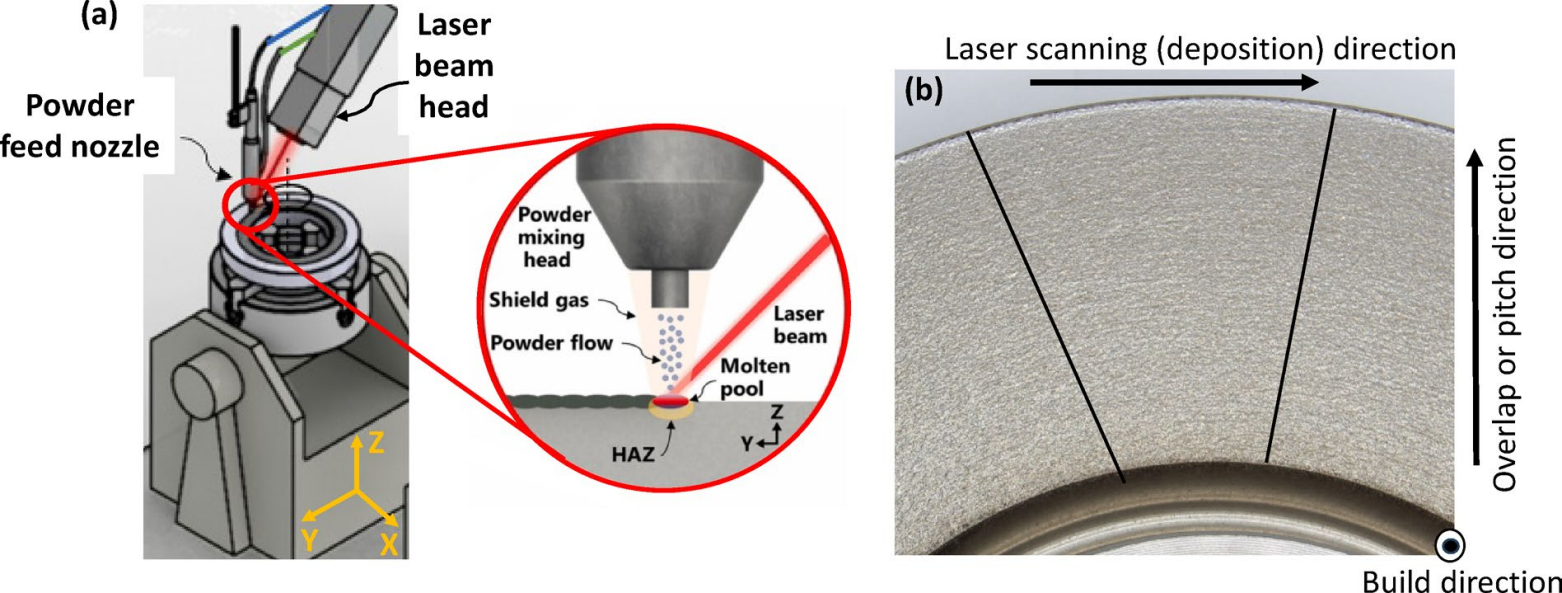
(**a**) Illustration of laser metal deposition of 316 L alloy annular disc (**b**) cut section of the deposited block showing laser scanning, overlap or pitch and build directions.

A 1-mm thick layer of AISI 316 L alloy was deposited onto an annular disc substrate made of mild steel G250 using direct laser energy deposition (DED). The deposition process followed a circular scanning pattern with an 80% overlap ratio and a radial pitch distance of 2 mm, starting from the center of the substrate. An illustration of the laser DED process is shown in Fig. 2.

The deposition experiments were conducted in a helium-argon gas flow chamber to prevent oxidation and contamination of the molten metal. The process parameters used during deposition are summarized in Table 1. To facilitate the process, the substrate was preheated to 100 °C using an oxyfuel torch.

The 316 L powders employed were water-atomized, with a mean particle size of approximately 110 μm and a range of 63 to 180 μm. Detailed powder morphology can be found in^19^. The chemical compositions of the 316 L alloy and the G250 substrate are summarized in Table 2.

The surface topography of the as-clad block was found to be notably rough, with a R_a_ value of 29 μm and an R_z_ value of 124 μm, which is very typical of additively manufactured surfaces. To remove the highly rough surface layer, the clad surface was ground using a surface grinding machine (BMT 4080 AH) with a diamond wheel at 1450 rpm. This was done in five passes, each removing a thin 5 μm layer, for a total removal of 30 μm from the top surface. The grinding aimed to minimize the introduction of further stress into the laser-clad surface prior to the subsequent post-processing by burnishing. The grinding was carried out along the laser cladding (scanning) direction, with cooling lubricant applied during the process. After grinding, the block was cut into coupons measuring 40 mm (L) x 40 mm (W) x 11 mm (H), and the ground surface was referred to as the “Ground” specimen.

**Table 1:** Laser direct energy deposition parameters used in this study.

Laser spot	Laser power	Laser scanning speed	Powder atomisation medium	Sheilding gas and flowrate	Carrier gas and flow rate	Nozzle stand-off distance	Pitching disance
4.8 mm	3.3 kW	1.8 m/min	Water	Helium and Argon (5 LPM)	Nitrogen (7 LPM)	17–19 mm	2 mm

**Table 2:** Chemical composition (wt%) of deded 316 L and substrate G250^20^.

Material	Fe	Cr	Ni	Mn	C	Si	*P*	S	Al
DEDed 316 L alloy	Bal.	14	9.6	1.01	0.02	0.3	0.01	0.01	-
Substrate G250	Bal.	-	-	0.25	0.07	0.03	0.02	0.02	0.07

### Ball burnishing surface treatment

**Fig. 3:**
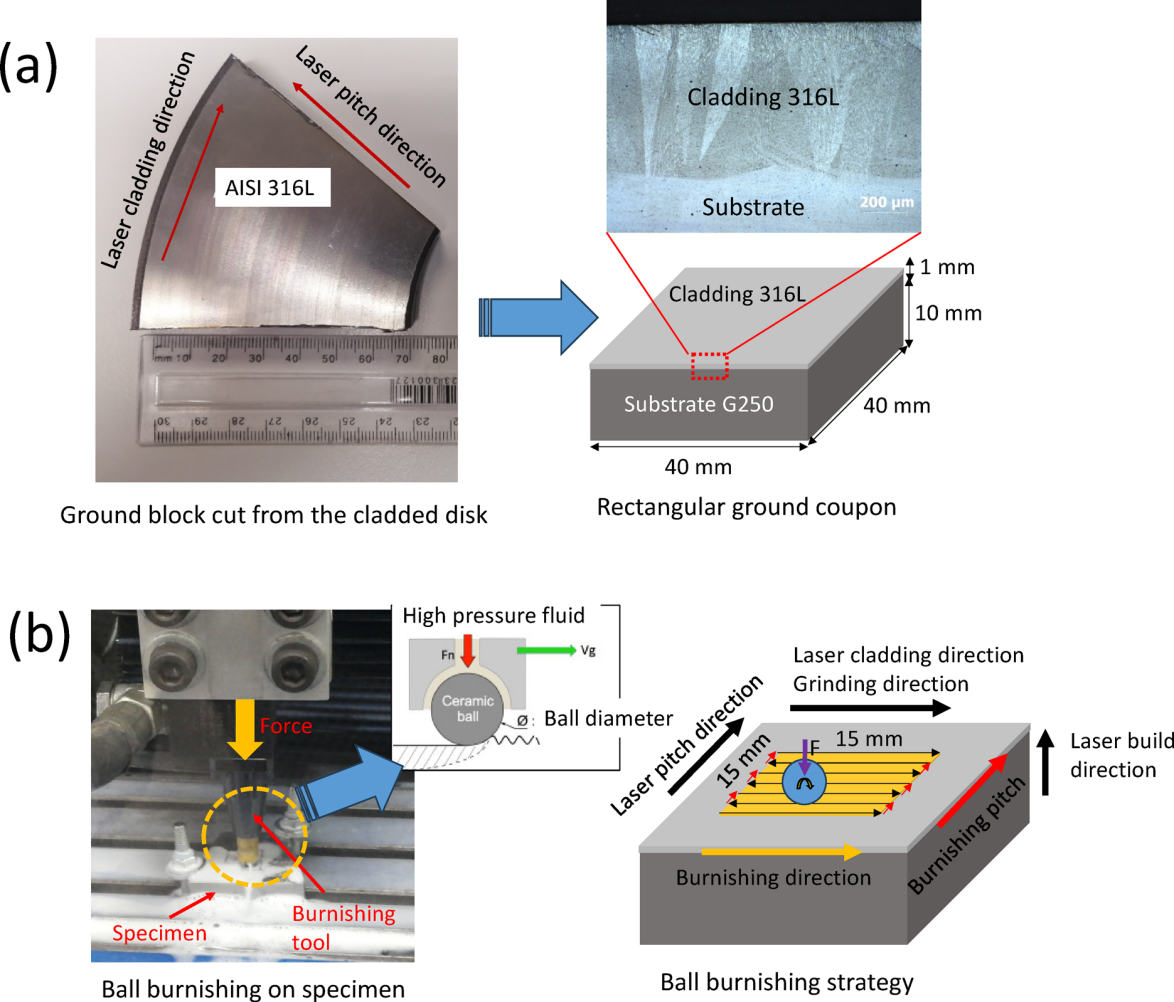
(**a**) Specimen preparation and (**b**) ball burnishing experimental setup and path strategy.

As is shown in Fig. 3, the burnishing was performed on a central 15 mm × 15 mm area of a rectangular ground coupon (40 mm × 40 mm × 11 mm) using a burnishing tool of HG6-9 E00° from Ecoroll. A hydraulically pressurized SiC (silicon carbide) ball, with a diameter of 6 mm, was employed to deform the material. The process was carried out at a hydraulic pressure of 160 bar, corresponding to a burnishing force of 452 N. The burnishing feed rate was set to 500 mm/min, with a constant step-over or pitch of 0.1 mm.

As shown in Fig. 3b, a zigzag continuous burnishing path strategy was adopted, with the burnishing direction aligned along the grinding or laser scanning direction, and the step-over perpendicular to the grinding direction. In Fig. 3b, it can be observed that hydraulic fluid was ejected through small holes in the ball insert, providing both lubrication and cooling at the interface between the burnishing ball and the specimen. The SiC burnishing ball, being significantly harder than the 316 alloy specimens, ensured that no permanent deformation occurred in the ball insert during the surface treatment. The specimen subjected to both grinding and burnishing was referred to as “Ground + Burnished.” Subsequent sections will focus on the comparison and discussion of surface integrity results between the “Ground” and “Ground + Burnished” specimens.

### Characterisations

#### Surface roughness

Surface roughness and topography were measured using a high-resolution optical confocal microscope (Alicona Infinite Focus), equipped with a 20x objective lens. The scanning region of interest (ROI) covered an area of 2.9 mm x 2.9 mm, with a sampling distance of 0.438 μm x 0.438 μm. The system’s cut-off filter, vertical resolution, and lateral resolution were set to 800 μm, < 50 nm, and 2.94 μm, respectively, to accurately capture the roughness features. Following the scan, form (plane) and surface waviness were removed using a suitable filter to minimize surface skewness errors. Both 2D profile roughness (Ra, Rz) and 3D surface roughness (Sa, Sz), along with a topographical image, were recorded. Profile surface roughness was measured perpendicular to the grinding line, laser cladding, and burnishing directions. Surface roughness measurements were taken at three different locations on each specimen coupon, with the average value calculated as the final result.

#### Microstructure

**Fig. 4:**
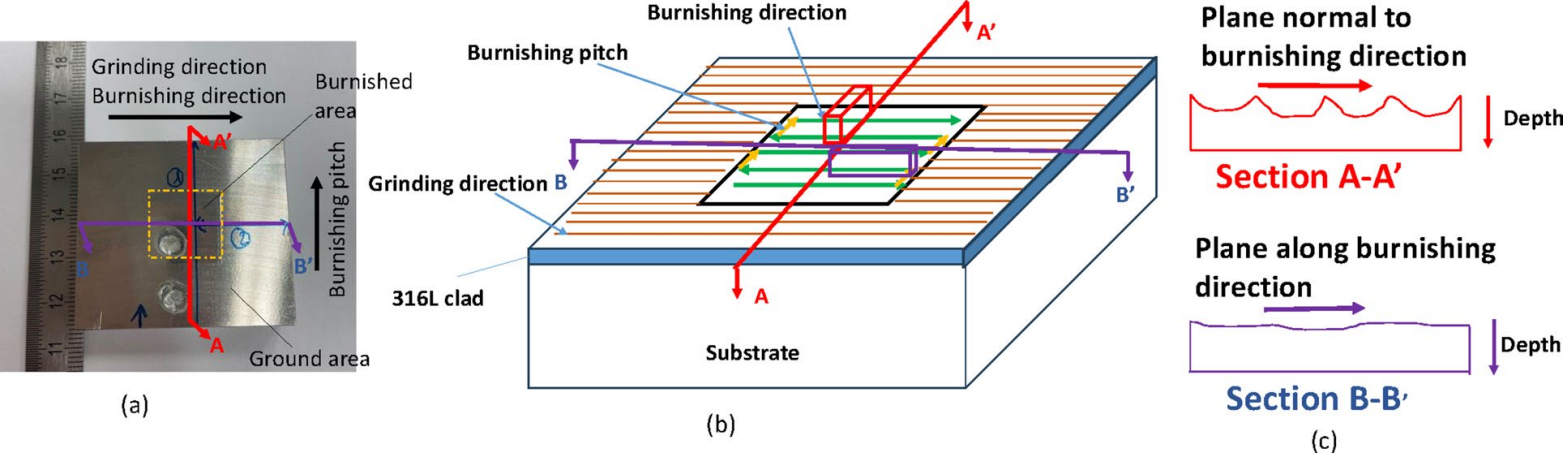
Illustration of sectioning of specimen for microstructural observation (**a**) actual burnished sample (**b**) cross-sectional planes along the burnishing and normal to burnishing directions, and (**c**) the schematic diagram of corresponding sectional surfaces along depth on which microstructural observations were made.

To examine the microstructural changes along and perpendicular to the burnishing direction, the specimen was cross-sectioned, ground, polished, and electro-etched. Figure 4 illustrates the sectioning of the specimen for microstructural observation. The cross-section along the depth, cut by the B-B’ plane, is referred to as the burnishing directional plane, while the section cut by the A-A’ plane is oriented normal to the burnishing direction, or along the burnishing pitch direction.

The specimen was initially cut using a manual saw cutting machine (Delta Abrasimet Cutter), equipped with a 250 mm diameter, 1.5 mm thick Al_2_O_3_ (alumina) cutter. The sectioned surface was then ground in stages with SiO2 sandpaper: P#240 for 2 min, P#600 for 2 min, and P#1200 with water for 4 min using BUEHLER’s AutoMet 300 machine, applying a grinding force of 20 N. The sample was polished on the same machine using a cloth pad with diamond particles of 9 μm for 2 min, followed by 3 μm diamond particles for another 2 min. Finally, alumina colloidal particles of 0.05 μm (pH 9.8) were used for 4 min to achieve a smooth, shiny, and crack-free surface.

To reveal the microstructures, the sample was electro-etched in a 4% (wt.) oxalic acid electrolyte at 5 V for 5 s. Microstructural images were captured using both Bruker’s TESCAN scanning electron microscope (SEM) and Zeiss’s AX10 HAL 100 optical microscope. Additionally, the energy dispersive x-ray spectroscopy (EDS) analysis to determine and confirm the appropriate chemical composition of the deposited layer on the substrate was performed on the same SEM instrument (Bruker’s TESCAN).

#### XRD

**Fig. 5:**
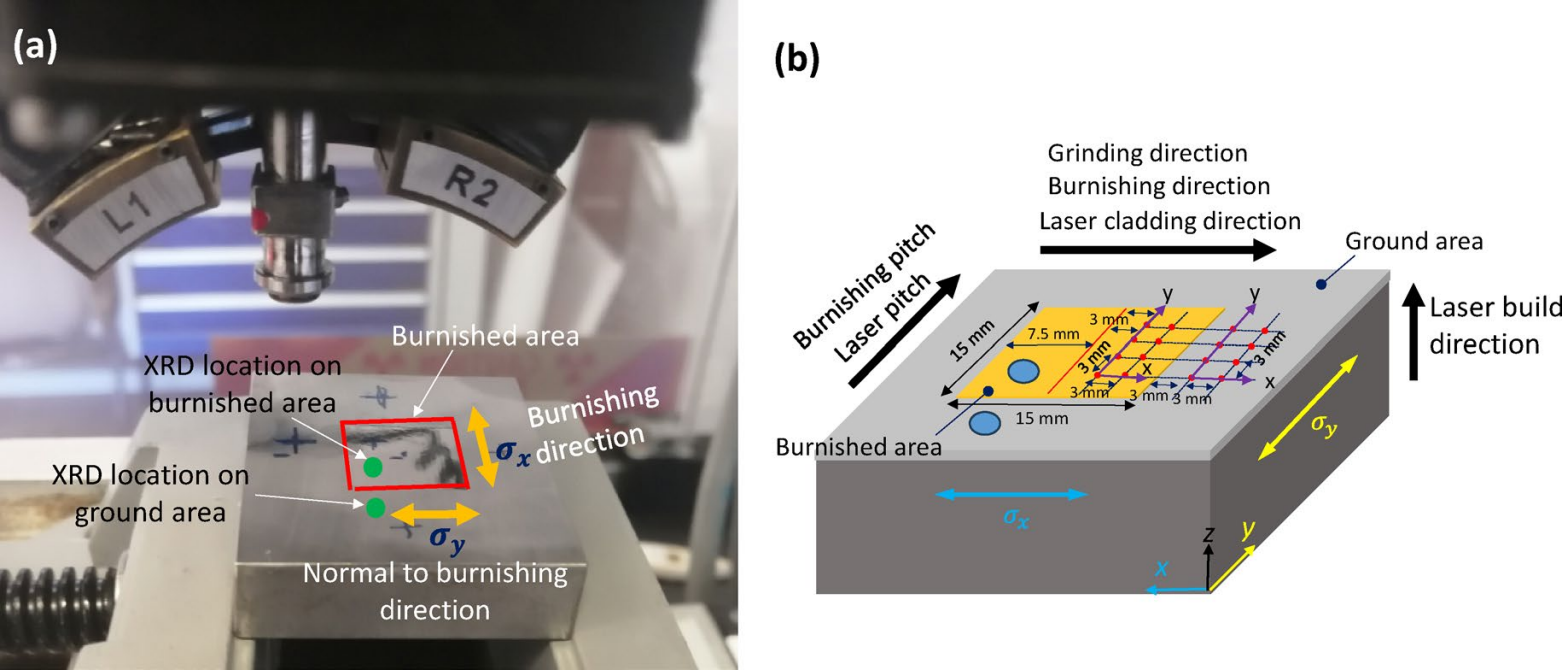
(**a**) XRD measurement setup (**b**) illustration of the array of locations for surface residual stress mapping.

Residual stress measurements were conducted on both ground and burnished specimens using Proto’s iXRD system. Figure 5 illustrates the XRD experimental setup and highlights the measurement locations on the sample. Residual stresses were evaluated in two directions: parallel to the burnishing direction (σ_x) and perpendicular to it (σ_y). Table 3 provides a summary of the measurement parameters used in this study.

To measure the in-depth residual stresses, material was removed layer by layer using Proto’s electrolytic polisher, operated at 45 V and 3.5 A in an ammonium chloride solution. After each layer was removed, the depth was measured using a Talysurf profilometer (Taylor Hobson). The amount of material to be removed was controlled by the electropolishing time. The Sin² Ψ vs. d-spacing method was employed to estimate the residual stress.

The X-ray diffraction measurements yielded peak breadths, also known as Full Width at Half Maximum (FWHMs). These peak breadths are primarily influenced by the grain size and dislocation density of the material under study.

**Table 3:** Residual stress measurement parameters.

Parameter	Value
Diffraction condition	Mn Kα x-ray tube 18 kV and 40 mA
Xray beam size	2 mm
Xray wavelength	2.103 Angstrom
Xray penetration	up to 5 μm
Bragg’s angle 2-theta	152.8°
Plane {hkl}	{311}
Bragg’s d-spacing	1.0819055 Angstrom
-S1(v/E)	1.20 E-6 [1/[MPa]
S2/2(1 + v)/E	7.18 E-6 [1/[MPa]
Acquisition	$\:\varPsi\:$ mode, 7 $\:\varPsi\:$ angles from − 30 to + 30°, exposure of 8 s

#### Micro-hardness

The microhardness was measured using Innovatest’s Falon 600 G2 hardness tester. The indenter was a diamond with a square pyramid shape and a point angle of 148°. Indentation was performed on the polished cross-sectional surfaces along the depth, starting from the top edge, as shown in the protocol in Fig. 4. This approach aimed to investigate the variation in hardness along two directions within the sample, induced by plastic deformation and material flow during burnishing.

On each surface, indents were made along three lines, with 80 μm spacing between each indent along the depth and 220 μm spacing between the lines. This resulted in a total of 31 data points per depth line, spanning approximately 2.48 mm, and a total of 91 indents (31 indents per line × 3 lines). The first indent was made 80 μm below the top edge of the surface. For each indent, the applied load was 0.1 kgf, with a holding time of 10 s.

## Results

### Surface roughness and topography

**Fig. 6:**
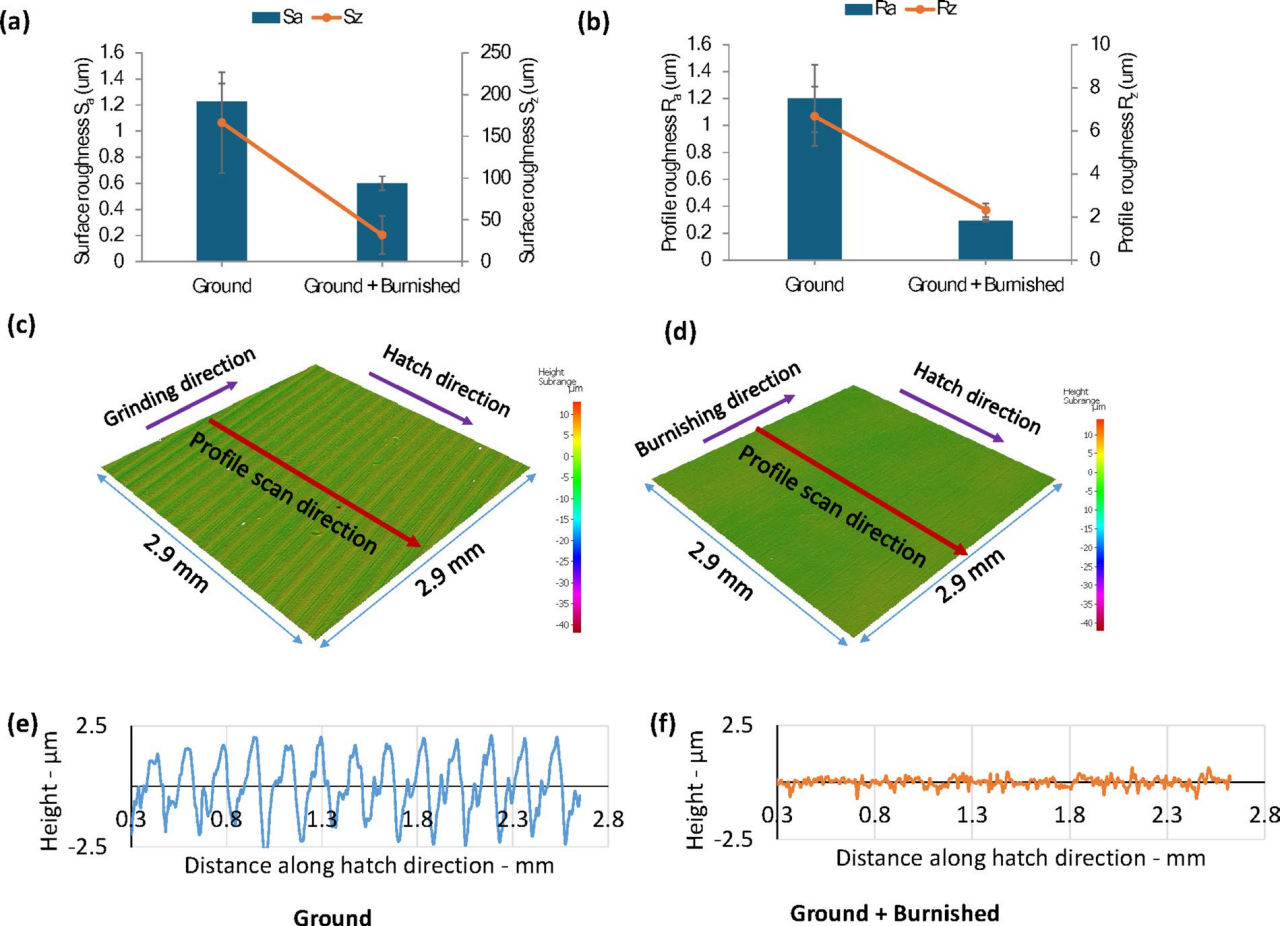
Comparison of surface roughness (**a**) Sa and Sz (**b**) Ra and Rz (**c,d**) 3D surface topography and (**e,f**) roughness profile between Ground and Ground + Burnished specimens.

As illustrated in Fig. 6, burnishing significantly enhanced the surface roughness of the ground surface. Specifically, burnishing reduced the Sa and Sz values by 51% and 81%, respectively, compared to the untreated ground surface (Fig. 6a). Additionally, Ra and Rz exhibited similar improvements, with reductions of 76% and 65%, respectively (Fig. 6b). The plastic deformation caused by the burnishing process resulted in the material flowing from the surface peaks into the valleys, creating a smoother and flatter topography (Fig. 6c, d). This effect is further demonstrated by the profile height scans taken perpendicular to the burnishing direction (Fig. 6e, f).

### SEM and EDS analysis

**Fig. 7:**
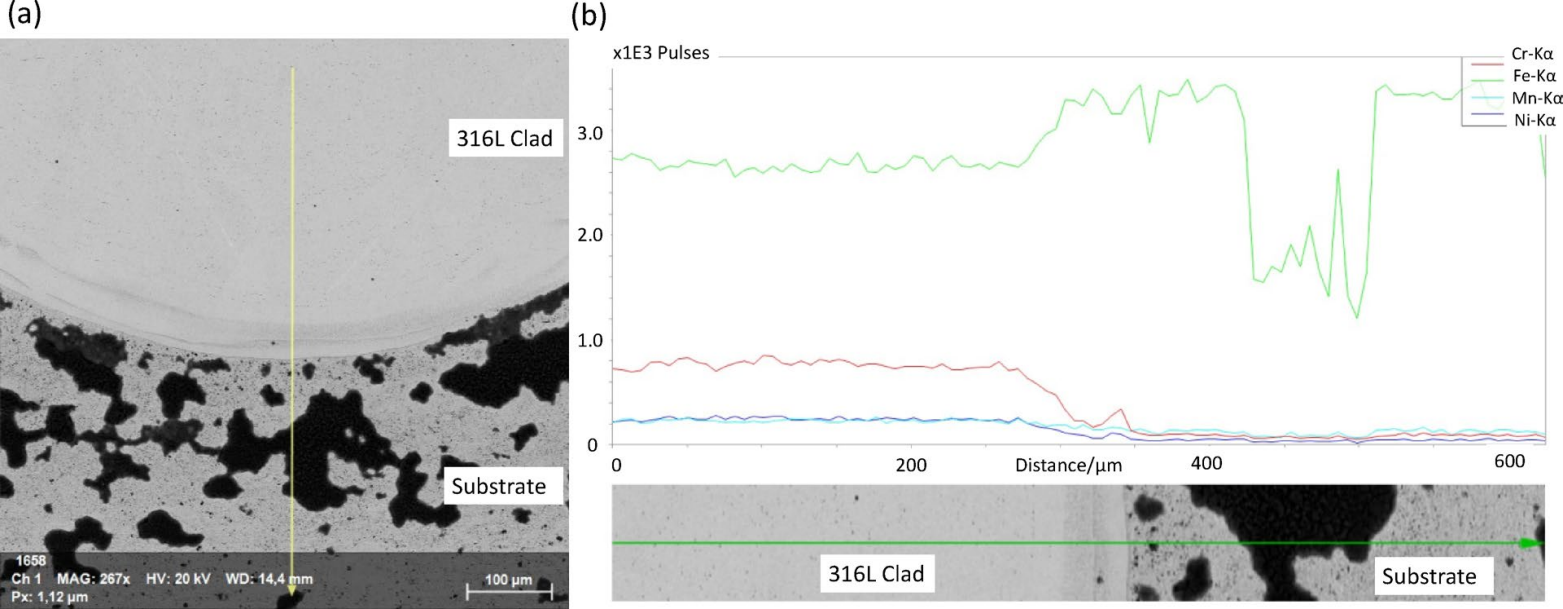
(**a**) Interface of 316 L cladding with G250 substrate and (**b**) their EDS profile showing the major chemical compositions. The yellow line arrow in (**a**) and the green line arrow in (b) are the EDS scan directions taken during measurement.

SEM and EDS analyses were conducted on the interface between the cladded 316 L and the substrate G250. As shown in Fig. 7, the cladded 316 L layer exhibited a high concentration of Fe, along with Cr, Mn, and Ni elements, while only Fe was detected on the substrate. The black areas observed on the substrate were identified as ferrite phases. This further confirms the successful laser direct energy deposition (LDED) of the 316 L coating, maintaining the specified elemental compositions.

**Fig. 8:**
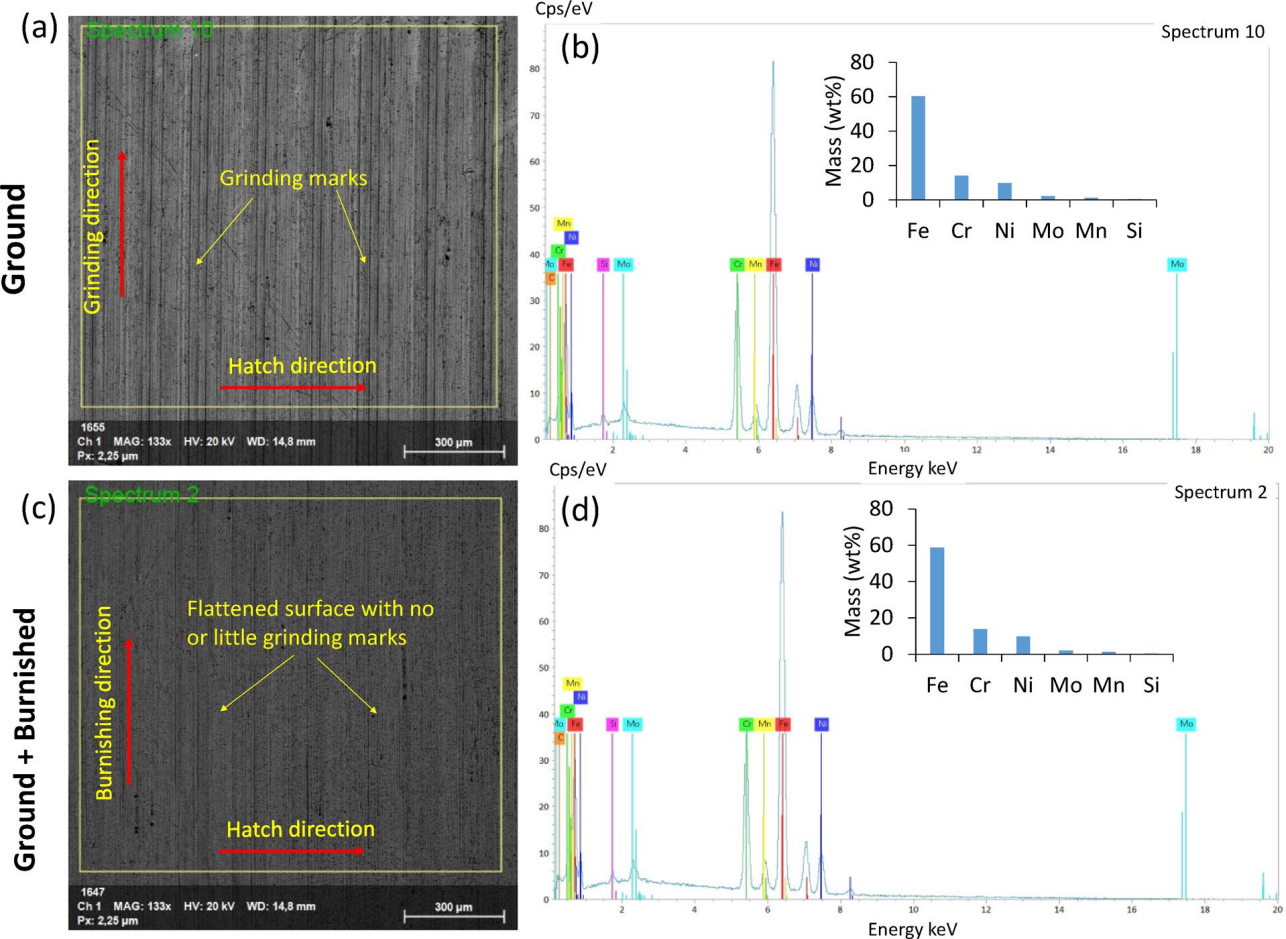
SEM image and EDS profile for (**a,b**) ground and (**c,d**) ground + burnished surfaces. The yellow squares in (**a,c**) define the surface areas over which the corresponding EDS spectrums were estimated. Inset bar plots in (**b,d**) show a comparison of total mass (%wt) of major relevant elemental compositions of the treated specimens.

Figure 8 presents SEM images of the surface topography for both the ground and burnished top surfaces. The ground surface displayed distinct linear grinding marks, accompanied by noticeable surface undulations. These characteristics are consistent with the findings of Abedini et al. (2024)^21^. Additionally, the combination of heat generation and the high-speed rotation of the abrasive wheel resulted in surface tearing and abrasive adhesion, as shown in Fig. 8 (a, c). In contrast, the burnishing effectively flattened and removed the grinding marks, leaving behind a smooth surface.

EDS spectra in Fig. 8 (b, d) show the corresponding surface chemical compositions. The mass (wt%) of the relevant elements for the 316 L coating remained consistent between the ground and ground + burnished surfaces, indicating that the burnishing did not introduce contamination. This finding aligns with the expected composition ratio for 316 L, which consists of Fe (60%), Cr (14%), Ni (10%), and Mo (2%) throughout the coating depth, as depicted in Fig. 7.

### Microstructural analysis

**Fig. 9:**
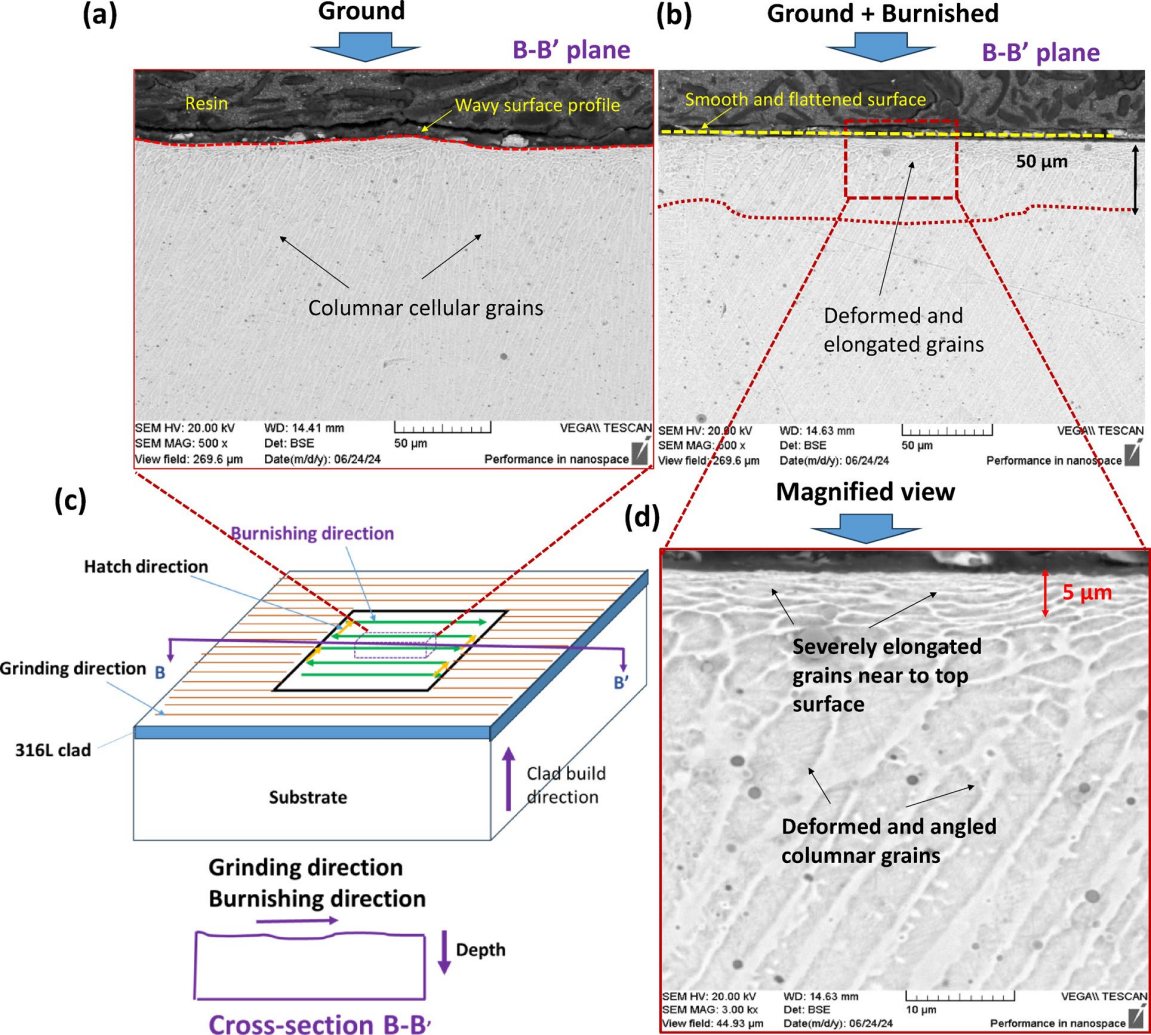
Comparison of microstructures of the treated surfaces (**a**) the ground, (**b**) ground + burnished samples, (**c**) illustration of cross-sectional plane along the burnishing/grinding direction (B-B’ plane) on which microstructures were observed, and (**d**) the magnified view of the microstructure for the ground + burnished sample in (**b**).

As illustrated in Fig. 9, the microstructures were examined to assess the impact of deformation and material flow along the burnishing direction (B-B’ plane). Columnar cellular grains were observed on both the ground and ground + burnished surfaces across the cross-sectional plane (as illustrated in Fig. 9c). This grain evolution is typical of the directional heating and cooling mechanisms inherent in the DED process. 316 L, an austenitic steel with a minimal ferrite content, primarily exhibited austenite phases, which appear as bright grey areas with white boundaries. While small surface pores were noted, the austenite phases predominated. As shown in Fig. 9a, the ground surface along the grinding (B-B’ plane) direction, displayed undulations or wavy profiles. However, the burnishing effectively flattened the undulated surface (Fig. 9b). After burnishing, the grains located within 50 μm of the top surface became deformed and elongated, and the extent of grain straining and shape alteration gradually diminished with increasing depth from the surface toward the bulk material. Importantly, highly deformed grains at a depth of 5 μm were observed as well (Fig. 9d).

**Fig. 10:**
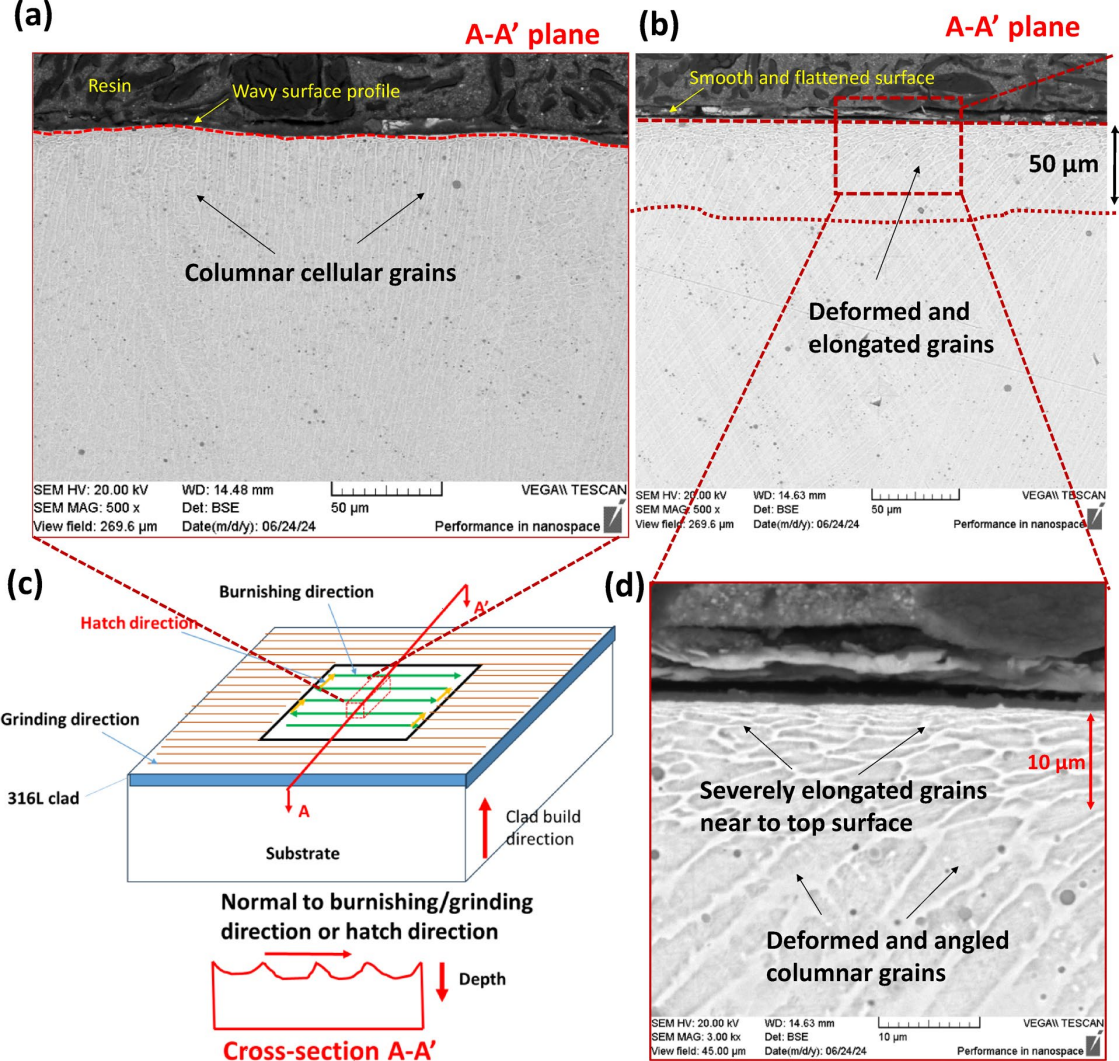
Comparison of microstructures of the treated surfaces (**a**) the ground, (**b**) ground + burnished samples, (**c**) illustration of cross-sectional plane along normal to the burnishing/grinding direction (A-A’) on which microstructures were observed, and (**d**) magnified view of the microstructure for the ground + burnished sample.

As shown in Fig. 10a, the ground surface along the normal to burnishing direction (A-A’ plane – Fig. 10c) showed a similar microstructure of columnar cellular grain growth along the build direction, while the top surface exhibited more waviness or undulations. However, the burnished surface on A-A’ plane revealed more equiaxed grains as deep as 10 μm beneath the surface, compared to a 5 μm depth of modified grain structure along the burnishing direction (B-B’ plane) (Fig. 10b, d). This discrepancy is due to more significant plastic deformation in the peaks of the ground surface during burnishing along the hatch direction, which caused material to flow into the valleys.

Overall, the burnishing induced grain straining and shape changes, potentially resulting in compressive residual stresses and increased hardness. It is also important to note that no grain refinement due to burnishing was observed in either of the directional cross-sectional surfaces (A-A’ and B-B’ planes). Grain refinement typically occurs through dynamic recrystallization under conditions of high plastic deformation and elevated temperature. In this study, the burnishing was conducted at a force of 452 N at room temperature, with ejected hydraulic fluid acting as a coolant at the interface. Consequently, the heat generated during burnishing is expected to be minimal, making any potential grain refinement insignificant. However, it is reported that the severe plastic straining causes increase in dislocation density or movement, which is related to the grain refinement as reported by many researchers including Wang et al. through 3D FEA and experimental investigation on laser shock peening on soft copper material^22^. Higher grain boundaries mean more dislocations, which is responsible for hardness improvement as presented in Sect. Micro-hardness analysis of the paper.

### Cross-sectional residual stress

**Fig. 11:**
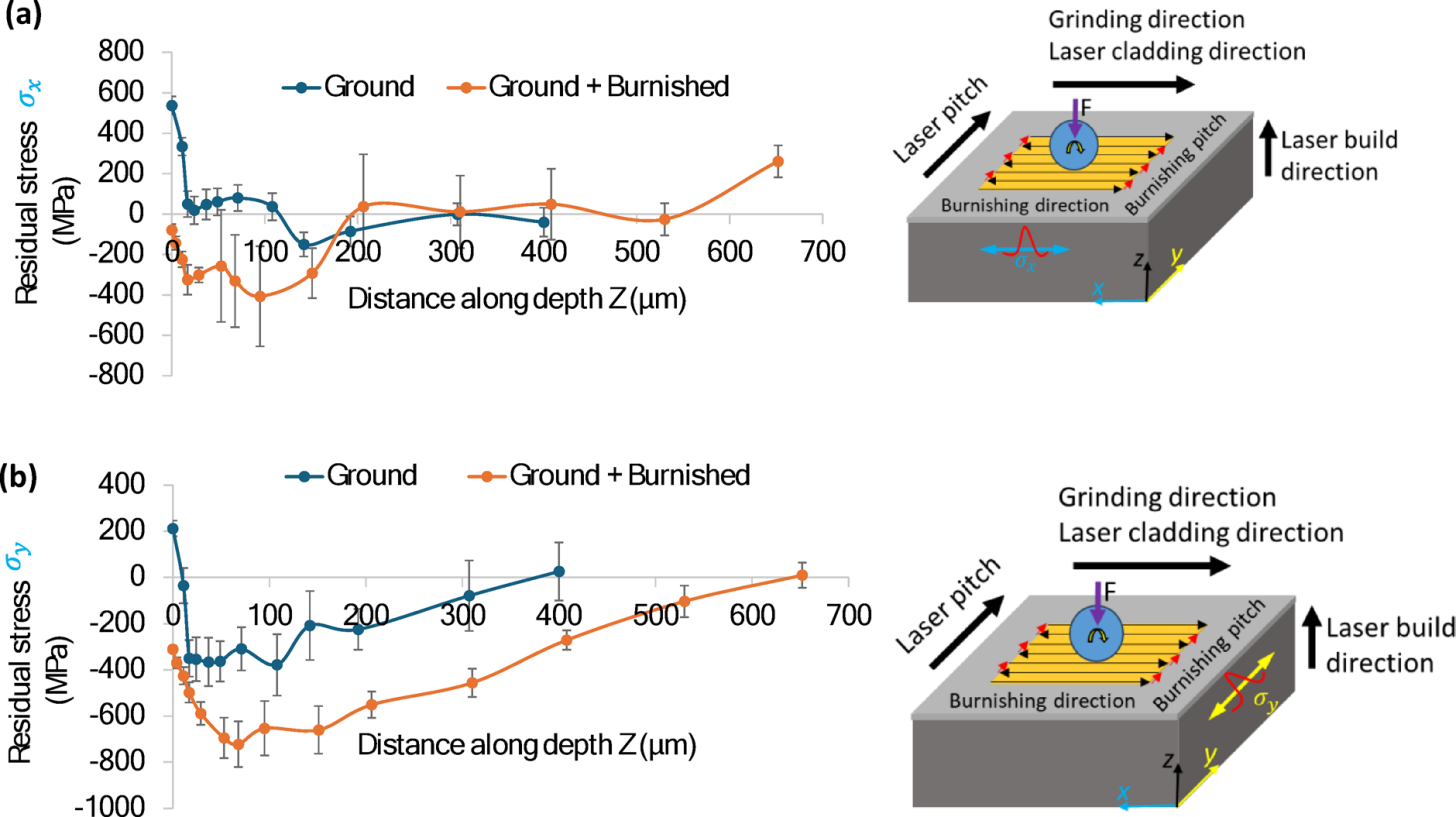
Comparison of residual stress with respect to distance along depth induced by burnishing along (**a**) the burnishing direction ($\:{\sigma\:}_{x}$) and (**b**) normal to burnishing direction ($\:{\sigma\:}_{y}$). Right side images in (**a,b**) depict an illustration of burnishing strategy and stress measurement directions.

Figure 11 presents a comparison of residual stress along the burnishing direction (σ_x_) and normal to the burnishing direction (σ_y_). As illustrated in Fig. 11a, the residual stress σ_x_ after grinding was tensile throughout the entire depth, extending from the top surface to 108 μm. However, following burnishing, σ_x_ became compressive, starting at -79 MPa at the top surface and increasing to a maximum of -405 MPa at a depth of 95 μm. Beyond this point, σ_x_ gradually transitioned back to tensile stress, with the total compressive stress layer extending to a depth of 200 μm.

In contrast, Fig. 11b shows the residual stress σ_y_ (normal to the burnishing direction). After grinding, σ_y_ was tensile at 212 MPa at the top surface, but reversed to compressive stress at 11 μm, reaching a maximum of -378 MPa at 108 μm. It then returned to tensile stress at a depth of 400 μm. Following burnishing, σ_y_ remained compressive, with a magnitude of -311 MPa at the surface, increasing to -722 MPa at 68 μm before decaying to tensile stress at a depth of 652 μm. These findings align with the increased depth of effective grain straining and modification along normal to the burnishing direction, as shown in Fig. 10. Thus, it is clear that the burnishing induces a deeper and more significant compressive stress in the normal direction (σ_y_) compared to the burnishing direction (σ_x_), due to the higher plastic flow of material in the burnishing pitch direction.

**Fig. 12:**
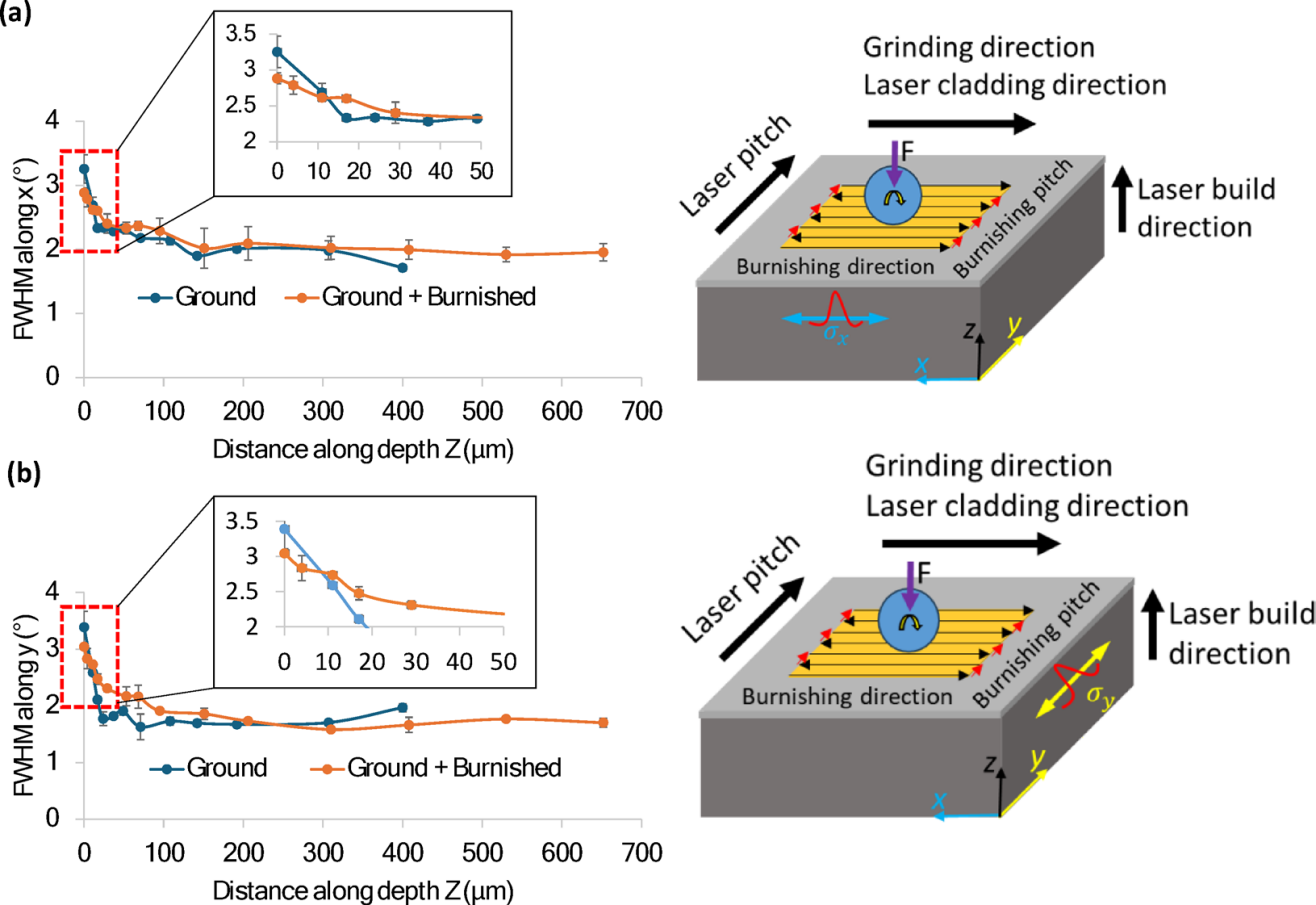
Comparison of FWHM with respect to distance along depth induced by burnishing along (**a**) the burnishing direction (x) and (**b**) normal to burnishing direction (y). Inset images in (**a,b**) show an illustration of burnishing and FWHM peak measurement directions.

The Full Width at Half Maximum (FWHM) serves as an indicator of grain straining, which is related to grain size. A higher FWHM corresponds to a smaller grain size. As shown in Fig. 12a, the FWHM trend along the x-direction (burnishing direction) remains consistent for both the ground and burnished specimens. The FWHM is highest at the top surface and decreases gradually with depth, stabilizing nearly constant throughout the bulk of the material. In contrast, the FWHM along the y-direction after burnishing is slightly higher than that of the ground surface, up to a depth of 200 μm, as shown in Fig. 12b. This suggests that burnishing induces greater and deeper grain straining along the burnishing pitch direction (y-axis). Due to plastic deformation in the burnishing pitch direction (y), the grains become more elongated, leading to smaller, more equiaxed shapes. Consequently, the X-ray diffraction (XRD) peaks broaden, and the FWHM increases.

An exception to this trend is highlighted in the magnified view (red dotted rectangular box) in the inset of Fig. 12, which shows that, from the top surface to a depth of 10 μm, the ground surface exhibits a higher FWHM along both the x and y directions compared to the burnished surface. However, beyond a depth of 10 μm, the effect of burnishing on FWHM becomes significantly more pronounced.

### Surface residual stress mapping

**Fig. 13:**
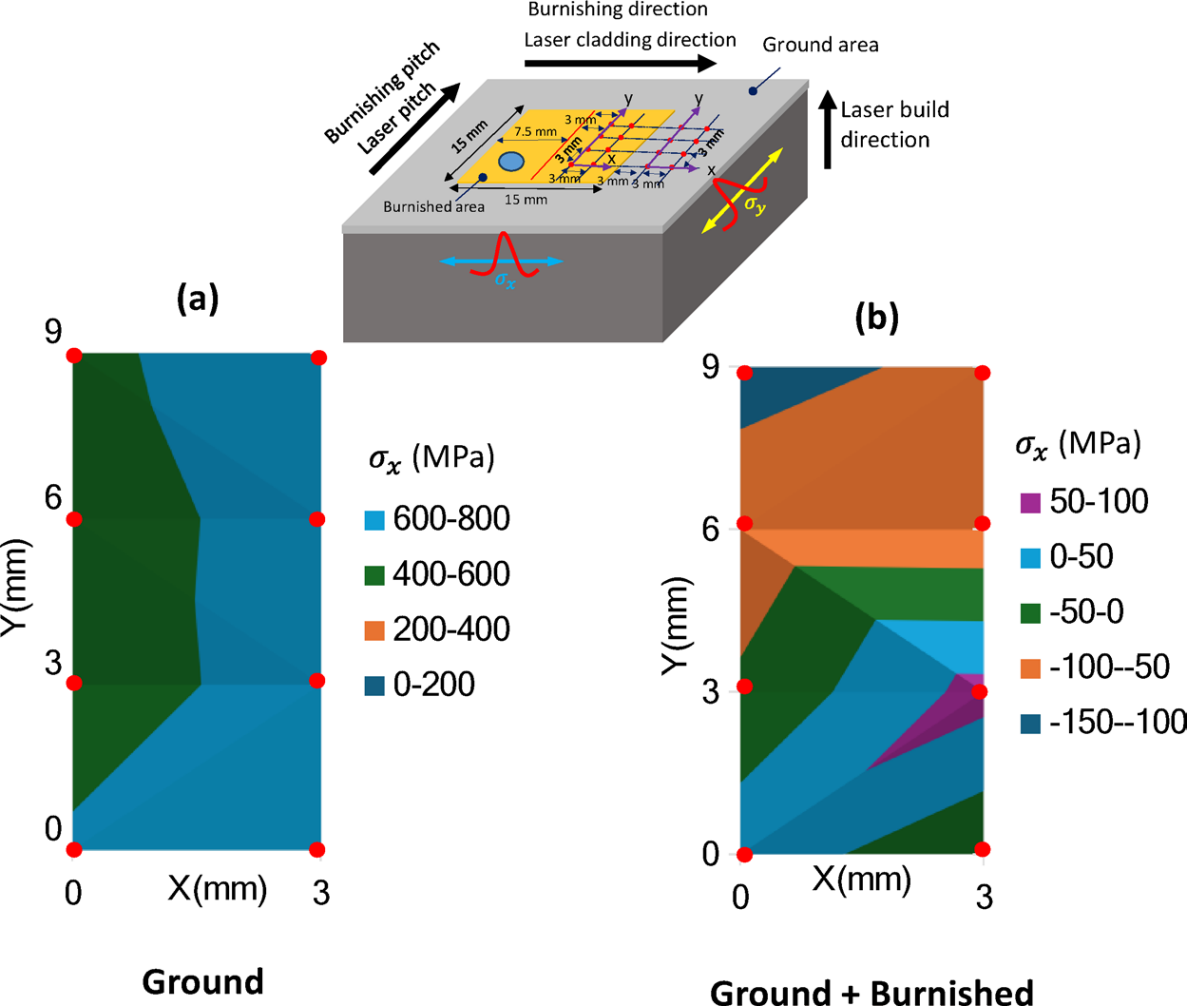
Mapping of surface residual stress $\:{\sigma\:}_{x}$ (along the burnishing direction) of (**a**) ground and (**b**) ground + burnished specimens. The inset photo shows burnishing and residual stress measurement locations and directions.

Figure 13 presents the mapping of surface residual stress, σ_x_ along the burnishing direction, over a 3 mm x 9 mm area. The data were collected at eight measurement points on both the ground and burnished surfaces, as shown in the inset photo. The measurements were taken 3 mm from the edge of the burnished surface boundary.

On the ground surface, σ_x_ was consistently tensile, with its magnitude varying between 600 and 800 MPa near the right edge of the specimen (Fig. 13a). In contrast, the burnished area primarily exhibited compressive stress, with values predominantly ranging from − 100 to -5 MPa towards the top of the region. However, some localized areas at the lower end of the burnished region displayed small tensile stresses, ranging between 50 and 100 MPa (Fig. 13b).

The tensile stress observed in certain regions could be attributed to localized stretching of the material at the surface peaks caused by the burnishing force. Notably, the compressive stress in the upper portion of the treated area was higher compared to the region where burnishing began (at the bottom-left corner). This increase in compressive stress could be a result of greater plastic deformation occurring as the burnishing process followed a zigzag path, progressing from the bottom-left to the upper-right region.

**Fig. 14:**
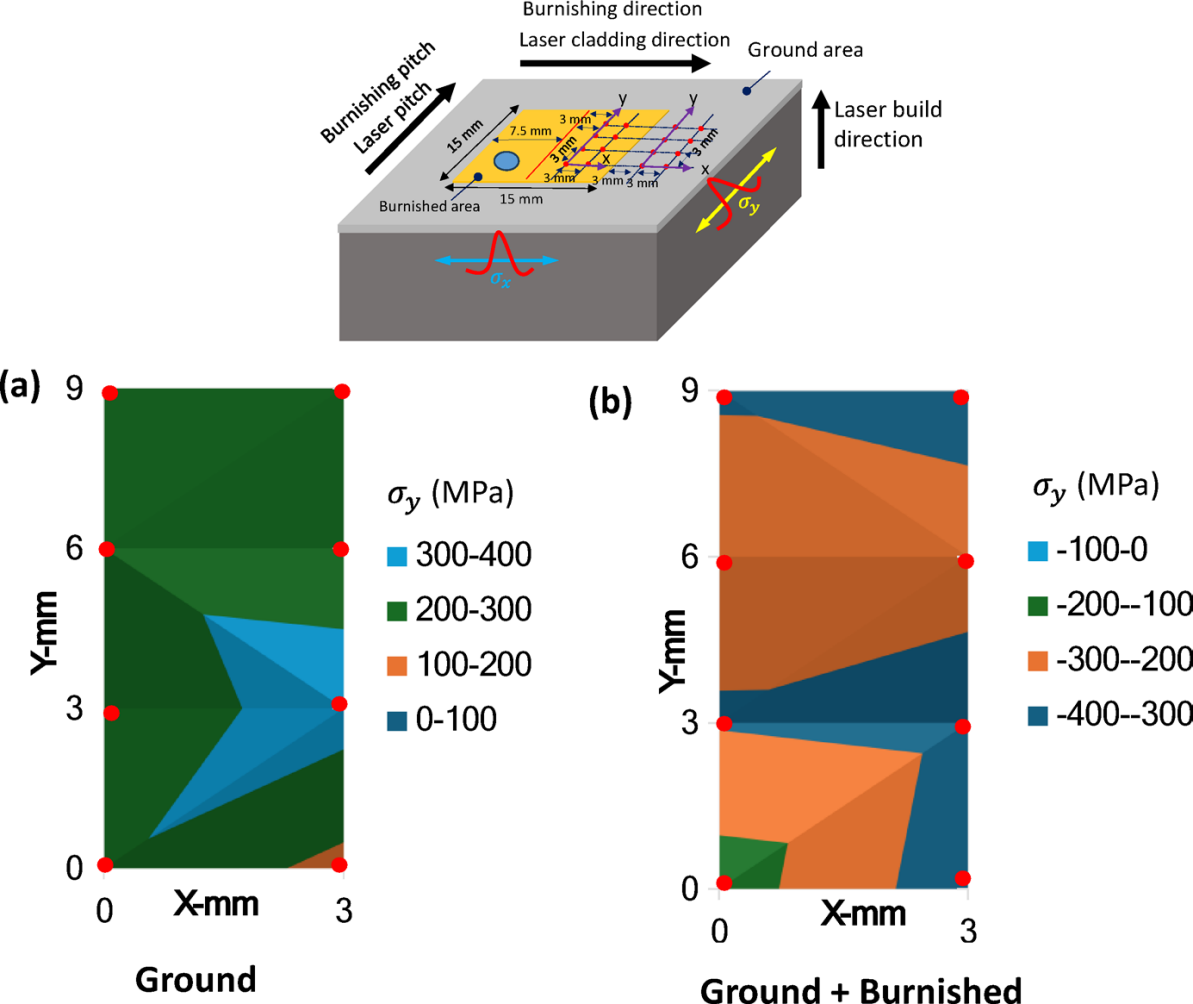
Mapping of surface residual stress $\:{\sigma\:}_{y}$ (normal to the burnishing direction) of (**a**) ground and (**b**) ground + burnished specimens. The inset photo shows burnishing and residual stress measurement locations and directions.

Figure 14 displays the mapping of surface stress σ_y_​ along the y-direction. Similar to σ_x_​, the magnitude of σ_y_​ on the ground surface was predominantly tensile, ranging from 200 to 300 MPa (indicated by the larger green region), with localized areas exhibiting higher tensile stress (light blue regions) (Fig. 14a). In contrast, the burnished surface exhibited a completely compressive stress field, with the majority of areas showing σ_y_​ values between − 200 and − 300 MPa (orange regions), and the highest variations occurring between − 300 and − 400 MPa (blue regions) (Fig. 14b). Furthermore, compressive stress σ_y_​ was found to be higher than σ_x_​. These results suggest that while grinding on the DED surface does not fully eliminate the detrimental tensile stress, plasticity burnishing successfully converted tensile stress into beneficial compressive stress across the surface. This transformation helps delay or minimize crack initiation and propagation, thereby preventing fatigue failure and enhancing the service life of DED components.

### Micro-hardness analysis

**Fig. 15:**
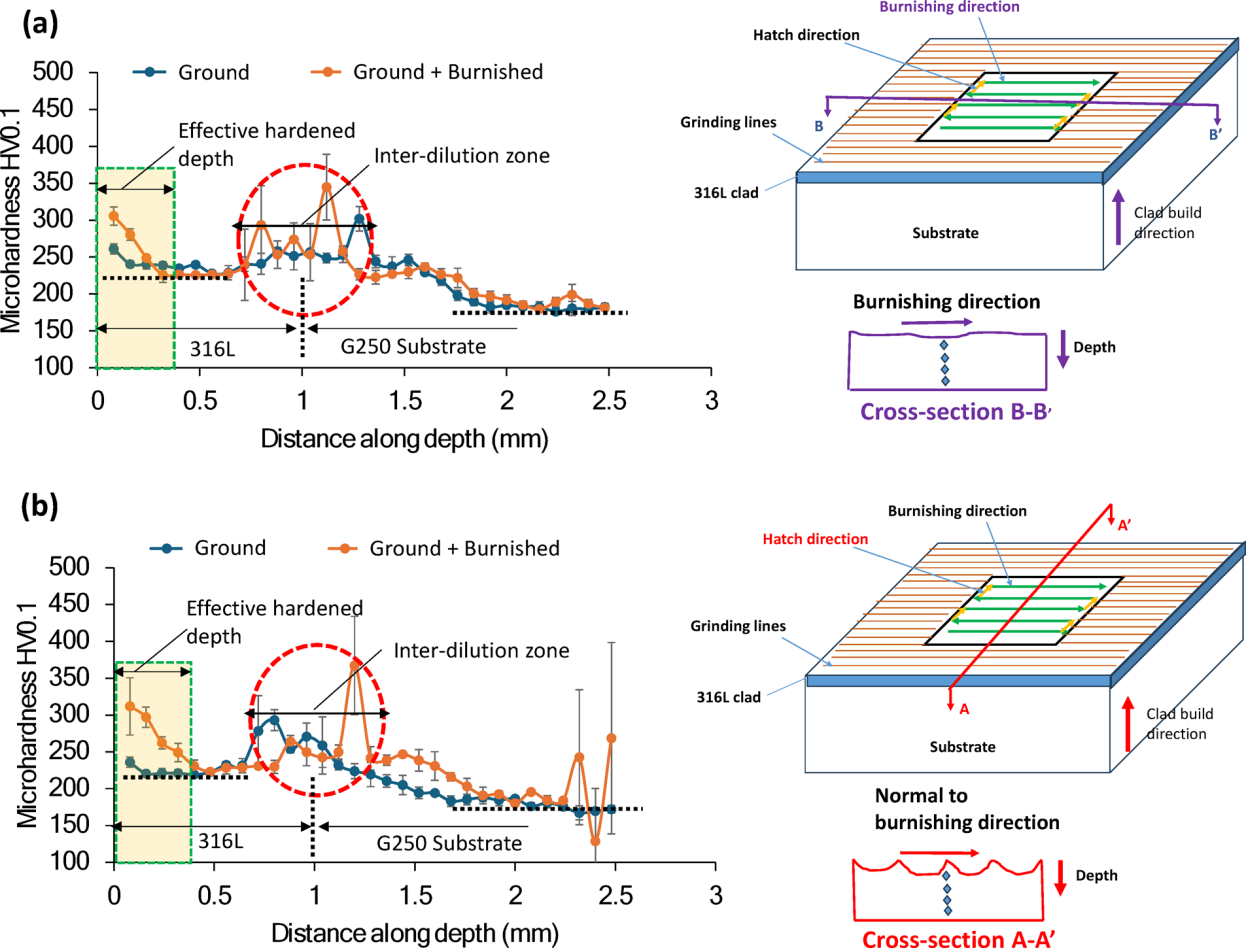
Comparison of microhardness on cross-sectional surfaces cut (**a**) along burnishing direction and (**b**) along normal to burnishing, as illustrated in schematic diagrams next to the hardness plots. Light yellow boxes in (**a,b**) highlight the effective modified depth of hardened material layer due to burnishing while the red circles indicate the sudden hardness increase in heat affected zone near the interface between the substrate and 316 L clad. Right side images indicate the cross-sectional planes on which the hardness was measured on the surface treated specimens.

Figure 15 presents a comparison of microhardness versus depth across cross-sectional surfaces along the burnishing direction (B-B’ plane) and normal to the burnishing direction (A-A’ plane). As shown in Fig. 15a, burnishing increased the hardness along the B-B’ plane by 17%, rising from 260 HV0.1 on the ground surface to 305 HV0.1 up to a depth of 400 μm. Beyond this depth, the hardness increase plateaued, aligning with the bulk hardness of 316 L. In contrast, Fig. 15b reveals that the hardness increase on the A-A’ plane (normal to the burnishing direction) was higher than on the B-B’ plane. Here, burnishing elevated the hardness by 32%, from 235 HV0.1 on the ground surface to 311 HV0.1, with the depth of modification remaining similar (approximately 400 μm) to that of the B-B’ plane. This greater hardness increase on the A-A’ plane is likely attributed to a more pronounced strain hardening effect along the burnishing pitch direction.

This phenomenon could be linked to the intrinsic characteristics of each deposition track and the overlap between them, which primarily dictates material plastic flow along the pitch direction. Thus, it can be inferred that hardness anisotropy may exist to some extent following ball burnishing of laser-cladded 316L. Additionally, the hardness distribution with depth aligns closely with the grain modification layer depth in the A-A’ and B-B’ cross-sectional planes shown in Figs. 9 and 10. A correlation between hardness and grain refinement due to burnishing or similar plastic surface modification processes has been documented in the literature^23^.

Furthermore, Fig. 15 also reveals distinct hardness peaks, indicated by the red dashed circles, at a depth of approximately 1 mm below the surface. In this study, the thickness of the laser-cladded 316 L layer was around 1 mm, and the burnishing force had negligible impact on these peaks. These hardness jumps are likely due to the hardened layer formed in the heat-affected dilution zone at the interface between the G250 substrate and 316 L cladding. This is a common phenomenon in DED processes. Beyond the dilution zone, the substrate G250’s hardness predominates.

## Discussion

Surface integrity plays a critical role in the functional performance of metal components. This study demonstrates that the surface integrity of DEDed 316 alloys can be enhanced through ball burnishing, making the parts suitable for practical applications. The directional effect of ball burnishing relative to the grinding or laser scanning direction of the DED surface is key to determining an effective post-processing strategy for plastic deformation of the material.

Our findings show that the burnishing significantly smooths the surface by removing grinding marks when performed in a direction perpendicular to the grinding or laser scanning direction. Additionally, greater grain modification depth, compressive stress, and hardness were observed on the cross-sectional surface along a plane normal to the burnishing direction (or hatch direction). This is due to the burnishing ball’s ability to push the peaks of the ground surface into the valleys through plastic deformation when burnishing perpendicular to the grinding lines. Furthermore, the smaller contact area between the ball and surface peaks results in higher plastic deformation, leading to greater grain misorientations and the development of more equiaxed grains that extend deeper into the material.

**Fig. 16:**
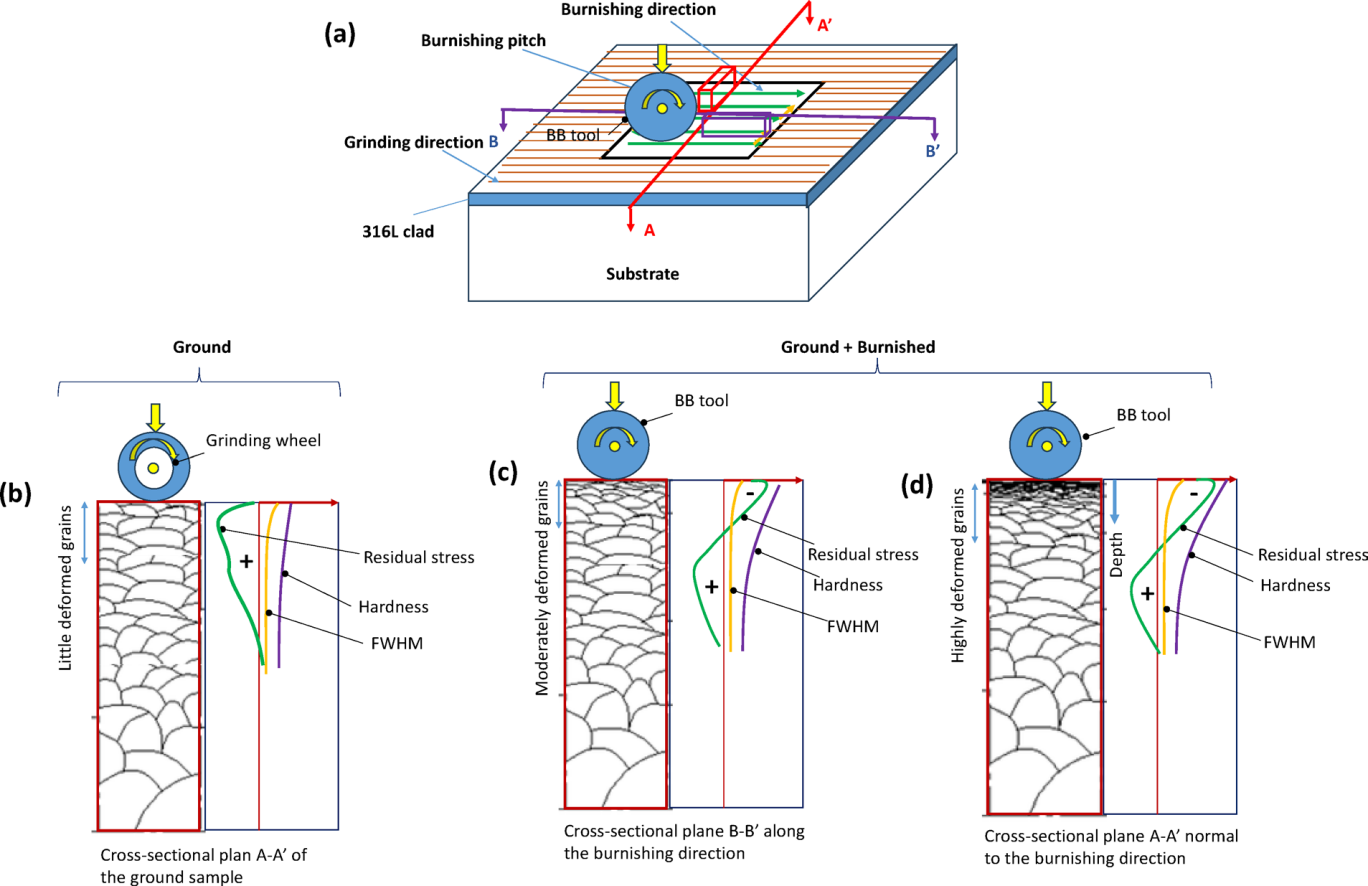
Illustration of plastically induced grain modification on the cross-section planes of the treated samples (**a**) definition of cross-sectional planes (**b**) ground sample A-A’ plane (**c**) ball burnished sample B-B’ plane (along the burnishing) and (**d**) ball burnished sample A-A’ plane (normal to the burnishing direction or burnishing pitch direction).

In contrast, when the burnishing ball follows the grinding lines, the ball tip primarily contacts the surface valleys, increasing the contact area and reducing the contact pressure. This leads to lower plastic deformation within the material. A comparative illustration of the grain modification from grinding and burnishing on cross-sectional planes—both along and perpendicular to the burnishing direction—is presented in Fig. 16.

Irrespective of the cross-sectional plane, the burnishing consistently increased compressive residual stress both on the top surface and along the depth of the material due to micro-straining of the grains. This grain modification mechanism aligns with the theory proposed by Williamson and Hall, which suggests that higher micro-strain results in higher compressive residual stress^24^. The correlation between increased micro-strain and higher full width at half maximum (FWHM) in the direction normal to the burnishing direction is evident in our results. These findings are consistent with the work of^23^who reported that slide and rotational burnishing induced higher compressive stress due to increased micro-straining within the material along normal to burnishing direction. Similar observation was noted by researchers in^7^. Moreover, this is aligned with our microstructural analysis as presented in Fig. 10 of Sect. Microstructural analysis.

As shown in Figs. 13 and 14, the burnishing not only generated higher compressive stress in deeper layers beneath the top surface but also resulted in localized tensile stress on the surface, which could potentially impact fatigue and corrosion resistance. Adjustments to burnishing force, feedback, pitch, and path strategy can help mitigate surface tensile stress and optimize performance.

Long columnar dendritic grain structures are a common feature in the Directed Energy Deposition (DED) process. After grinding, there is little apparent change in the grain structure. The grinding force is relatively low, insufficient to cause significant alterations in grain orientation. As a result, the ground specimen exhibits a uniformly distributed pattern of similar, longer grains throughout the depth of the cross-sectional surface. Although the heat generated during grinding softens the material, the rapid cooling that follows induces surface residual tensile stress. This tensile stress is particularly pronounced on the top surface of the ground specimen, which may contribute to the initiation and propagation of fatigue failure. A similar phenomenon regarding grain evolution due to grinding in DED-produced 718 alloys was further investigated and confirmed in a study by Qi et al. (2024)^23^.

## Conclusions

This paper investigates the effect of burnishing on the surface integrity and residual stress of DEDed 316 L alloys, with a particular focus on characteristics changes in surface properties across two directional planes relative to the burnishing direction. The major findings of the study are summarized as follows:

(1) The grinding effectively removed the rough layer of the DEDed specimen, while the ball burnishing further enhanced the surface finish by plastically deforming the surface peaks and valleys. However, little traces of smeared surface marks remained visible, indicating the potential need for additional post-processing.

(2) The burnishing altered the microstructure beneath the surface, transforming grains from a cellular/columnar to an equiaxed shape. This effect was most pronounced in the plane normal to the burnishing direction, extending to a depth of 50 μm, whereas the plane aligned with the burnishing direction showed less significant modification.

(3) The burnishing proved highly effective in converting tensile stress into compressive stress. The maximum in-depth compressive stress was observed on the plane normal to the burnishing direction (σ_y_ = -722 MPa at 68 μm), which was significantly higher than that along the burnishing direction (σ_x_ = -407 MPa at 95 μm). Additionally, the burnishing resulted in an increase in the full width at half maximum (FWHM) of XRD peaks along normal to the burnishing direction (y), with the highest FWHM observed at a depth of 68 μm.

(4) Unlike the predominantly tensile stress observed on the top surface of the ground specimen, the burnished surface exhibited mostly compressive stresses along both the burnishing direction (σ_x_) and normal to it (σ_y_), with maximum variations ranging from − 100 to -400 MPa. Localized tensile stresses (σ_x_ = 50–100 MPa) were still present on the burnished surface, which could be addressed either through further post-processing or by adjusting the burnishing parameters.

(5) Thanks to grain modification and potential dislocation movements, the burnishing led to an increase in microhardness by up to 32% at the top surface, with the hardened layer extending approximately 400 μm in depth. Hardness improvements were more pronounced in the cross-sectional plane normal to the burnishing direction than along the burnishing direction.

In conclusion, the burnishing exhibits a directional effect on material deformation, resulting in variations in the surface integrity of DEDed 316 L alloys. This is primarily influenced by the contact pressure and the interaction between the burnishing tool and the surface peaks and valleys. The degree of deformation and surface integrity is also dependent on the initial surface roughness and the burnishing processing parameters. The findings of this study offer valuable insights for developing burnishing path strategies, enabling tailored surface integrity and functional performance for DEDed 316 L alloys in real-world applications.

## Data Availability

The XRD datasets generated and/or analyzed during the current study are available in the UniSA Sharepoint at “https://mymailunisaedu-my.sharepoint.com/:f:/g/personal/uddinms_unisa_edu_au/EuooxUnZmttAjYksw_sIy_oB9ZbG12Y033aKWwUSx1yDAA? e=img3Rg” and “raw data files 316L”. Further queries/data available from the corresponding author on reasonable request.
